# Prognostic role of HPV integration status and molecular profile in advanced anal carcinoma: An ancillary study to the epitopes-HPV02 trial

**DOI:** 10.3389/fonc.2022.941676

**Published:** 2022-10-14

**Authors:** Alice Debernardi, Aurélia Meurisse, Jean-Luc Prétet, David Guenat, Franck Monnien, Laurie Spehner, Angélique Vienot, Patrick Roncarati, Thierry André, Laurent Abramowitz, Chloé Molimard, Christiane Mougin, Michael Herfs, Stefano Kim, Christophe Borg

**Affiliations:** ^1^ EA3181, University of Bourgogne Franche-Comté, LabEx LipSTIC ANR-11-LABX-0021, Besançon, France; ^2^ Methodology and Quality of Life in Oncology Unit, University Hospital of Besançon, Besançon, France; ^3^ INSERM, EFS BFC, UMR1098 RIGHT, University of Bourgogne Franche-Comté, Besançon, France; ^4^ Papillomavirus National Reference Center, University Hospital, Besançon, France; ^5^ Molecular Biology and Microbiology Department, Anamed SA Laboratory, Lausanne, Switzerland; ^6^ Department of Pathology, University Hospital of Besançon, Besançon, France; ^7^ Department of Medical Oncology, University Hospital of Besançon, Besançon, France; ^8^ Laboratory of Experimental Pathology, GIGA-Cancer, University of Liege, Liege, Belgium; ^9^ Department of Medical Oncology, University Hospital Saint Antoine, Paris, France; ^10^ Division of Gastroenterology and Hepatology and Proctology, University Hospital Bichat, Paris, France; ^11^ Ramsay GDS, Blomet Clinic, Paris, France; ^12^ Department of Anatomopathology, University Hospital of Besançon, Besançon, France; ^13^ Clinical investigation center, CIC-1403 University Hospital of Besançon, Besançon, France; ^14^ Department of Medical Oncology, Sanatorio Allende, Cordoba, Argentina

**Keywords:** NGS - next generation sequencing, SCCA, Somatic mutation analysis, TERT promoter mutation, HPV integration

## Abstract

Squamous Cell Carcinoma of the Anal canal (SCCA) is a rare disease associated with a Human Papillomavirus (HPV) infection in most cases, predominantly the HPV16 genotype. About 15% of SCCA are diagnosed in metastatic stage and some will relapse after initial chemoradiotherapy (CRT). Treatment of patients by Docetaxel, Cisplatin and 5-fluorouracil (DCF) has been recently shown to improve their complete remission and progression-free survival. The aim of this retrospective study was to explore the impact of HPV infection, HPV DNA integration, TERT promoter mutational status and somatic mutations of oncogenes on both progression-free (PFS) and overall survivals (OS) of patients treated by DCF. Samples obtained from 49 patients included in the Epitopes-HPV02 clinical trial, diagnosed with metastatic or non-resectable local recurrent SCCA treated by DCF, were used for analyses. Median PFS and OS were not associated with HPV status. Patients with episomal HPV had an improved PFS compared with SCCA patients with integrated HPV genome (p=0.07). *TERT* promoter mutations were rarely observed and did not specifically distribute in a subset of SCCA and did not impact DCF efficacy. Among the 42 genes investigated, few gene alterations were observed, and were in majority amplifications (68.4%), but none were significantly correlated to PFS. As no biomarker is significantly associated with patients’ survival, it prompts us to include every patient failing CRT or with metastatic disease in DCF strategy.

## Introduction

Squamous cell carcinoma of the anal canal is a rare disease, representing less than 3% of all gastrointestinal malignancies in the world (1). Its incidence has been steadily increasing in recent decades in men as well as in women with 50 000 new cases diagnosed each year worldwide and it is estimated that it will continue to increase in the next future (2). This increase is likely due to its association with human papillomavirus infection (3), predominantly genotype HPV16, since HPV-related oncoproteins (E6 and E7) are expressed in more than 90% of patients with SCCA (4).

The great majority of SCCA patients are diagnosed at a localized stage. However, about 15% of patients are diagnosed at metastatic stage in US (https://seer.cancer.gov/statfacts/html/anus.html, accessed July 25^th^ 2022), and between 25% to 40% of patients treated initially by chemoradiotherapy will develop locally advanced recurrences or metastases in Western countries (5–7). Treatment of patients with nonresectable local recurrences or with distant metastases relies on systemic chemotherapy. The combination of cisplatin (CDDP) and 5-Fluorouracil (5FU) was historically considered as the recommended treatment for advanced SCCA based in retrospective analysis (8, 9). However, complete remission was a rare event, and only about 15% of patients were progression-free at 1 year (10, 11).

Docetaxel is an anticancer agent which exerts cytotoxic functions by stabilizing tubulin polymerization leading to mitosis arrest and cell death. It has been previously proposed that a loss of normal p53 function confers sensitization to taxane chemotherapy by increasing G2/M arrest and apoptosis (12). Because the association between SCCA and HPV infection is strong and E6 oncoprotein encoded by High Risk (HR) HPV, such as HPV16 and 18, induces the degradation of p53, we previously hypothesized that SCCA might be sensitive to taxane-containing chemotherapies such as docetaxel (13). In addition, docetaxel has been shown to increase endoplasmic reticulum (ER) stress and to induce immunogenic cell death of cancer cells (14). In 2018, Epitopes-HPV02 trial confirmed the benefit of the addition of docetaxel to CDDP and 5FU (DCF) (15). A complete response was observed in 45% of patients, and the 1-year progression free survival rate was 47%. Therefore, DCF became one of the standard regimen at first-line in advanced SCCA (13, 15–17).

Here, we describe the molecular characterization of anal cancer biopsies with advanced SCCA, included in the Epitopes-HPV02 trial.

## Material and methods

### Patients and cell lines

The cohort is constituted of patients included in the prospective multicenter phase II Epitopes-HPV02 study (NCT02402842). Patients with diagnosis of metastatic or non-resectable local recurrent SCCA were included, and were treated by DCF. The study was described in detail elsewhere (15–17). Among 66 patients included in the trial, 49 patients had available material for molecular analysis. The EDITH V cohort constituted of patients with diagnosis of SCCA at early stage was used as a validation cohort. The study was described elsewhere (18). Overall, 381 patients had available material for molecular analysis.

### DNA extraction

Prior to DNA extraction, separate hematoxylin-eosin-stained slides were reviewed by an experienced histopathologist and manually macro-dissected when appropriate to ensure tumor content greater than 20%. Depending on the size of the fixed tissue, between 3 and 8 formalin-fixed paraffin-embedded (FFPE) tissue sections of 10 µm thickness were processed for DNA extraction with the QIAamp DNA mini kit (QIAGEN) according to manufacturer’s instructions.

### HPV genotyping

HPV genotyping was performed locally [Department of Cellular and Molecular Biology, University Hospital of Besançon, France as described previously (19)]. Briefly, genotyping was performed with the INNO-LiPA HPV Genotyping Extra^®^ test (Fujirebio) allowing the identification of 28 different HPV genotypes as well as the HLA-DPB1 gene as internal control for sample quality for DNA detection. As recommended by the manufacturer, samples negative for the HLA-DPB1 gene and negative for HPV were excluded from the analysis.

### *In situ* hybridization

To confirm the HPV infection, *in situ* hybridization experiments were performed according to the manufacturer’s instructions. The INFORM HPV III Family 16 Probe (Ventana Medical Systems) allowed the detection of 12 high-risk HPV genotypes.

### HPV physical status

As routinely performed, the physical status of HPV (episomal or mixed/integrated) was determined by assessing the disruption of the viral *E2* gene (**Table 1**). Briefly, after DNA extraction and concentration measurement with NanoDrop 1000 spectrophotometer (Thermo Fisher Scientific), quantitative real-time PCR experiments were performed as follows: 95°C for 15 min, 40 cycles at 95°C for 30 sec, then 50°C for 1 min and 72°C for 1 min. Each experiment was performed in triplicate and the E6/E2 ratio cut-off value was determined, as previously described (20, 21). Importantly, to be able to compare the collected results, the amplification efficiency of each PCR reaction was determined (qPCR efficiency calculator, Thermo Fisher Scientific).

**Table 1:** Primer sequence used for *E2*, *E6* and *GAPDH* qPCR.

Primer	Sequence
HPV16 *E2* Fw	5’- TTTAGCAGCAACGAAGTATCC-3’
HPV16 *E2* Rev	5’- AGTCTCTGTGCAACAACTTAG-3’
HPV16 *E6* Fw	5’- AAAGCCACTGTGTCCTGAAGA-3’
HPV16 *E6* Rev	5’-CTGGGTTTCTCTACGTGTTCT -3’
*GAPDH* Fw	5’-ACCAGGTGGTCTCCTCTGAC-3’
*GAPDH* Rev	5’-TGCTGTAGCCAAATTGGTTG-3’

### *TERT* mutations analysis by Sanger sequencing

Genomic sequence of promoter flanking region of *TERT* (ENSR00001274355) was obtained from Ensembl database (www.ensembl.org). Specific primers were designed (**Table 2**) using the online Primer-BLAST software (www.ncbi.nlm.nih.gov/tools/primer-blast/) (22). Targeted sequences were amplified by PCR using the Qiagen Multiplex PCR kit (QIAGEN) and 5% DMSO. PCR conditions were as follows: 94°C for 15 min, 40 cycles at 94°C for 1 min, then 64°C for 30 sec, 72°C for 45 sec and finally 7 min at 72°C. PCR products were purified using the gel extraction kit NucleoSpin Gel and PCR Clean-up (Macherey-Nagel). Bidirectional sequencing reaction was performed using the BigDye Terminator v3.1 Cycle Sequencing Kit (Life technologies-Thermofisher). The reactions were run according to the following protocol: one cycle at 96°C for 1 min; 15 cycles at 96°C for 10 s, 50°C for 5 s, 60°C for 1 min 15 s; 5 cycles at 96°C for 10 s, 50°C for 5 s, 60°C for 1 min 30 s; 5 cycles of 96°C for 10 s, 50°C for 5 s and 60°C for 2 min. After purification with a NucleoSEQ kit (Macherey-Nagel), samples were run and analyzed on an ABI 3130 sequencer (Life technologies-Thermofisher). Finally, the sequences obtained were compared with the reference sequence of *TERT* promoter using GeneScan analysis software.

**Table 2:** Primer sequence used for *TERT* sequencing.

Primer	Sequence
*TERT* Fw	5’-CGTCCTGCCCCTTCACCT-3’
*TERT* Rev	5’-AGCGCTGCCTGAAACTCG-3’

### *TERT* mutations analysis by SNaPshot

SNaPshot analysis was performed from the purified amplicons used for Sanger sequencing with the ABI Prism SNaPshot Multiplex kit (AB Life Technologies). Amplified *TERT* promoter was analyzed for the presence of mutations at position C228 and C250 using two primers that contained an additional poly(dC) tail at their 5’ end, allowing for their simultaneous detection (**Table 3**). Reactions were performed in a final volume of 5 µL, containing 1.5 µL of purified multiplex PCR product (2 to 10 ng/µL), 2.5 µL of SNaPshot Ready Multiplex Ready Reaction Mix, 0.5 µL of probe equimolar mix (each probe at 0.2 pmol/L final), and 0.5 µL of double-distilled water. Multiplex single base extensions were carried out for 25 cycles according to the following program: 10 seconds at 96°C, 5 seconds at 52°C, and 30 seconds at 60°C. SNaPshot products were then treated at 37°C for 15 min with 0.5µL of shrimp alkaline phosphatase at 1 U/µL diluted in 2.5 µL of shrimp alkaline phosphatase buffer 10X and 11.5 µL of double-distilled water. After heat inactivation of shrimp alkaline phosphatase for 10 minutes at 75°C, 2 µL of the labelled products were mixed with 9.5 µL of HiDi formamide and 0.5 µL of Genescan-120LIZ size standard. They were then separated using a 25 min run on an ABI Prism 3130 DNA sequencer with POP-7 matrix and 14 seconds for injection. The analysis was performed using GeneMapper ID software version 3.2.1 (Applied Biosystems).

**Table 3:** SNaPshot primers used for detection of *TERT* promoter mutation.

Primer	Sequence	Size (bp)	Mutation
C228	5’-T_23_GGCTGGGAGGGCCCGGA-3’	40	C228T
			C228A
C250	5’-T_39_CTGGGCCGGGGACCCGG-3’	56	C250T

### Libraries preparation and NGS

Libraries were prepared from 50 ng of DNA or by using KAPA Hyperplus Library Preparation (KAPA Biosystem) and Solid Tumor Solution capture kits and protocol by SOPHIA GENETICS. They were sequenced on MiSeq sequencer (Illumina). Criteria used to select mutations were depth (≥100) and allele frequency (≥10%). Allele frequency variants ≈100% or described as benign in ClinVar database, with intronic, frameshift, splicing or synonymous mutations were excluded. A list of the targeted 42 genes is shown in **Table 4**.

**Table 4:** List of the genes and their exons targeted by NGS.

Gene	Exon	Gene	Exon
*AKT1*	3	*HIST1H3B*	1
*ALK*	21-25	*HRAS*	2-4
*BRAF*	11,15	*IDH1*	4
*CDK4*	2	*IDH2*	4
*CDKN2A*	1*,2,3	*KIT*	8-11,13,17,18
*CTNNB1*	3	*KRAS*	2-4
*DDR2*	18	*MAP2K1*	2,3
*DICER1*	24,25	*MET*	2,14-20
*EGFR*	18-21	*MYOD1*	1
*ERBB2*	8,17,20	*NRAS*	2-4
*ERBB4*	10,12	*PDGFRA*	12,14,18
*FBXW7*	8-12	*PIK3CA*	2*,3,6*,8,10,21
*FGFR1*	13,15	*PTPN11*	3
*FGFR2*	7,12,14	*RAC1*	3
*FGFR3*	7,9,14,16	*RAF1*	7,10,12,13*,14*,15*
*FOXL2*	1*	*RET*	11,13,15,16
*GNA11*	4,5	*ROS1*	38*,41*
*GNAQ*	4,5	*SF3B1*	15-17
*GNAS*	8	*SMAD4*	8-12
*H3F3A*	2*	*TERT*	promoter*,1*,8*,9*,13*
*H3F3B*	2*	*TP53*	full coding region

A dash (-) means “from exon X to exon X”. A star (*) means hotspots only.

### Statistical analysis

Log-rank (Mantel-Cox) test was used for the analysis of PFS and OS according to HPV integration status and *PIK3CA* mutational status. PFS was determined at 12 months from the first DCF cycle. Fisher’s exact test was used for all other analysis.

## Results

### Influence of HPV status and integration in SCCA patients included in Epitopes HPV02 study

Among patients included in the Epitopes-HPV02 study, there was a majority of female (83.7%) compared to male who represented only 16.3% (**Table 5**). Most of the patients (91.8%, 45 out of the 49 patients with available tumor material) presented an HPV16 infection. Two patients were infected with other HPV genotypes (HPV33 and HPV33-45) and two patients had SCCA without detectable HPV genome. Median PFS was 10.7 months (95% CI: 9.9-16.0) for patients displaying HPV16^+^ SCCA and 12.6 months (95% CI: 6.2-18.9) when HPV was not detected. Median OS was 36.3 months (95% CI: 24.2-NE) for HPV16^+^ SCCA and 26 months for HPV negative SCCA.

**Table 5:** Characteristics of the HPV02 cohort.

HPV02 cohort (n=49)	
**Sexe**	
Female	83.7% (n=41)
Male	16.3% (n=8)
**HPV status**	
HPV16	91.8% (n=45)
Other HR-HPV	4.1% (n=2)
HPV negative	4.1% (n=2)
**Integration status**	
Episomal	28.6% (n=14)
Integrated/mixed forms	51% (n=25)
NR or HPV negative	20.4% (n=10)
**PFS (months)**	
Median	10.7
Min	1.8
Max	42.6
**OS (months)**	
Median	33
Min	3.8
Max	46.9

**Figure 1:**
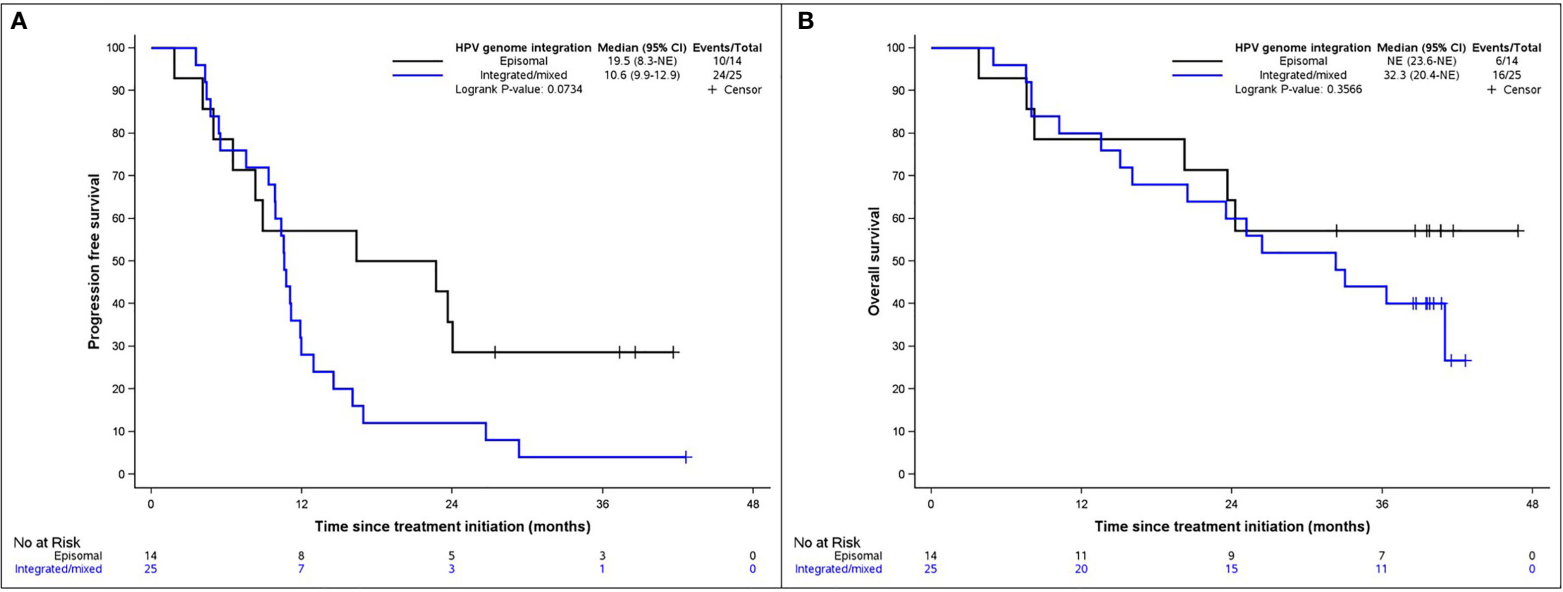
Kaplan-Meier diagrams representing **(A)** PFS and **(B)** OS according to the integration of HPV genome. Blue line symbolizes integrated/mixed forms, black line episomal forms.

We next explored if the presence of HPV genome in an episomal or integrated form could influence clinical outcomes of SCCA patients treated with DCF (**Table 6**). There was a majority of integrated/mixed forms (51%, n=25) in all SCCA compared to episomal forms (28.6%, n=14). In about 20% (n=10/49, 20.4%) of patients, the HPV integration status was not determined (2 of them were actually HPV negative, 2 were infected with other HR-HPV infection than HPV16 and the others had no remaining material to perform analysis). Median PFS was 19.5 months (95% CI: 8.3-NE) for patients with HPV DNA under episomal form and 10.6 months (95% CI: 9.9-12.9) for patients with integrated/mixed forms (p=0.0734). Median OS was 32.3 months for patients with integrated/mixed forms and was not reached for SCCA with episomal HPV DNA (**Figure 1**). The possible correlation between episomal HPV DNA in DCF efficacy is outlined by the 57% complete response rate observed in this population vs 44% for SCCA wherein HPV DNA was integrated or in a mixed form.

**Table 6:** Clinical characteristics of SCCA patients according to HPV genome status.

	Overall population n=39	Episomal n=14	Integrated/mixed n=25	p-value
**Gender**				
Male	7 (17.9%)	2 (14.3%)	5 (20%)	1
Female	32 (82.1%)	12 (85.7%)	20 (80%)	
**Age**				
Mean (std)	57.5 (8.4)	58.6 (6.6)	56.8 (9.3)	
Median (min-max)	58.2 (38.6-74.9)	59.8 (46.1-71.7)	56.1 (38.6-74.9)	0.6318
Q1-Q3	51.4-63.8	52.1-63.8	49.4-63.7	
**Age ≥ 65**				
No	32 (82.1%)	13 (92.9%)	19 (76%)	0.3863
Yes	7 (17.9%)	1 (7.1%)	6 (24%)	
**ECOG**				
0	27 (69.2%)	9 (64.3%)	18 (72%)	0.7232
1	12 (30.8%)	5 (35.7%)	7 (28%)	
**HIV positivity**				
No	38 (97.4%)	14 (100%)	24 (96%)	1
Yes	1 (2.6%)	0 (0%)	1 (4%)	
**T**				
Missing (or x)	8	3	5	
0	1 (3.2%)	1 (9.1%)	0 (0%)	0.0590
1	1 (3.2%)	0 (0%)	1 (5%)	
2	13 (41.9%)	3 (27.3%)	10 (50%)	
3	7 (22.6%)	1 (9.1%)	6 (30%)	
4	9 (29%)	6 (54.5%)	3 (15%)	
**N**				
Missing (or x)	11	5	6	
0	8 (28.6%)	3 (33.3%)	5 (26.3%)	0.4424
1	5 (17.9%)	0 (0%)	5 (26.3%)	
2	5 (17.9%)	2 (22.2%)	3 (15.8%)	
3	10 (35.7%)	4 (44.4%)	6 (31.6%)	
**M**				
Missing	8	3	5	
0	21 (67.7%)	6 (54.5%)	15 (75%)	0.4232
1	10 (32.3%)	5 (45.5%)	5 (25%)	
**RTCT**				
No	15 (38.5%)	8 (57.1%)	7 (28%)	0.0727
Yes	24 (61.5%)	6 (42.9%)	18 (72%)	
**Surgery**				
No	35 (89.7%)	13 (92.9%)	22 (88%)	1
Yes	4 (10.3%)	1 (7.1%)	3 (12%)	
**Stage**				
Locally advanced	4 (10.3%)	1 (7.1%)	3 (12%)	**0.0351**
Synchronous metastasis	12 (30.8%)	8 (57.1%)	4 (16%)	
Metachronous metastasis	23 (59%)	5 (35.7%)	18 (72%)	
**Invaded sites**				
Mean (std)	2.4 (1.3)	2.1 (0.9)	2.6 (1.4)	
Median (min-max)	2 (1-5)	2 (1-4)	2 (1-5)	0.3314
Q1-Q3	01-mars	01-mars	01-mars	
**Invaded sites**				
1	11 (28.2%)	4 (28.6%)	7 (28%)	0.5195
2	12 (30.8%)	6 (42.9%)	6 (24%)	
3	9 (23.1%)	3 (21.4%)	6 (24%)	
≥ 4	7 (17.9%)	1 (7.1%)	6 (24%)	
**Best L1 response**				
Complete response	19 (48.7%)	8 (57.1%)	11 (44%)	0.4310
Partial response	18 (46.2%)	5 (35.7%)	13 (52%)	
Stability	1 (2.6%)	0 (0%)	1 (4%)	
Progression	1 (2.6%)	1 (7.1%)	0 (0%)	

### Distribution of *TERT* promoter mutations in SCCA patients

**Figure 2:**
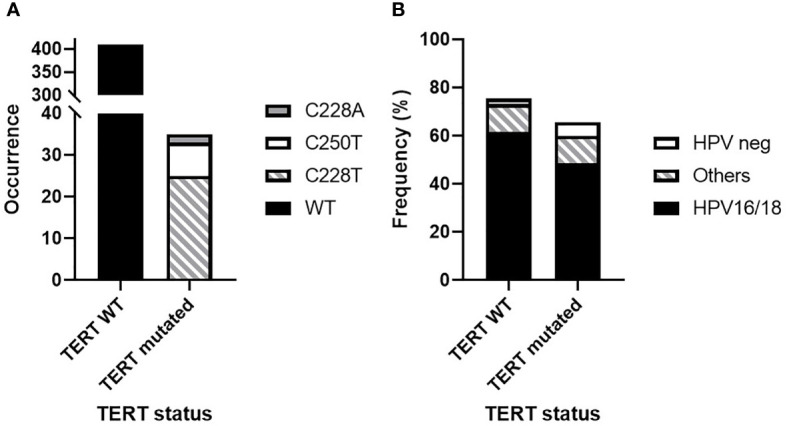
*TERT* promoter mutation distribution in human SCCA from the HPV02 and EDITH V cohorts (444 samples). **(A)** Wild-type (WT) *TERT* promoter prevalence versus C228T, C228A and C250T mutations. **(B)** Distribution of HPV status according to *TERT* promoter mutations.

Transactivation of TERT is a critical signaling for HPV-mediated oncogenesis. E6-E6AP ubiquitin ligase complex are known to bind to *TERT* promoter, activating *TERT* gene transcription (23). Mutations of the *TERT* promoter are the major genomic alterations leading to TERT overactivation in most cancer types. However, the occurrence of *TERT* promoter mutations was never investigated in SCCA. We hypothesized that *TERT* promoter mutations might sustain resistance to DCF therapy. Therefore, these analyses were performed in 63 patients with available tumor materials. *TERT* promoter mutations occurred rarely and were observed in 5 patients (7.9%). Three types of *TERT* promoter mutations investigated have been observed. One HPV16^+^ SCCA patient had a C250T mutation, and another HPV16^+^ had a C228A mutation. Three other patients (2 HPV16^+^ and one HPV^-^ SCCA) had C228T mutations. Three of the SCCA patients displaying *TERT* promoter mutations showed partial responses after exposition to DCF. These results addressed the question of the overall distribution of *TERT* mutations in SCCA population. To validate the prevalence of *TERT* mutations in SCCA, sequencing analysis of the telomerase catalytic subunit *TERT* was performed in the EDITH V cohort. Among the 381 patients with available DNA, *TERT* promoter mutations were identified in 30 patients (7.8%). The C228T mutation was predominant (73.3%, n=22), while the mutations C228A and C250T were observed in 1 and 7 patients respectively. Of note, one homozygote mutation of C228T was detected. Among the EDITH V cohort, there was a majority of patients with HPV positive SCCA (68%, n=259) including 210 (81%) patients with HPV16 or 18 infections and 49 (19%) patients with other HPV genotypes; 10 (2.6%) patients represented HPV negative SCCA. HPV status was not available for 112 (29.4%) patients. *TERT* promoter mutations were not specifically correlated to HPV status in SCCA (**Figure 2**). These results showed that the rare *TERT* promoter mutations observed in SCCA are not restricted to HPV negative SCCA and are not specifically correlated to a specific HPV type infection.

### Distribution of other gene mutations in SCCA patients

Since HPV genotype, integration status or *TERT* promoter mutations did not account for DCF efficacy and SCCA patients’ prognosis in the Epitopes-HPV02 study, we next assessed the distribution of the main oncogenic alterations in SCCA metastatic or relapsing patients treated by DCF. NGS analysis targeting 42 oncogenic alterations was realized among patients of the HPV02 cohort. No alterations were found in *AKT1, ALK, BRAF, DDR2, DICER1, ERBB4, FOXL2, GNAQ, GNAS, H3F3A, H3F3B, HIST1H3B, IDH1, IDH2, KIT, KRAS, MAP2K1, MET, PDGFRA, PTPN11, RAC1, RET, ROS1, SF3B1, SMAD4* and *TP53. PIK3CA* was the most altered gene with 10 amplifications and 6 mutations, followed by *ERBB2* (3 amplifications and 3 mutations) and *CDK4* (5 amplifications) (**Table 7**).

**Table 7:** Summary of somatic mutations retrieved among SCCA from the HPV02 cohort.

Gene	Exon	Alteration	Protein	Occurrence
*PIK3CA*		Amplification		n=10
* *	10	c.1633G>A	p.Glu545Lys	n=6
*ERBB2*		Amplification		n=3
* *	17	c.1960-1963delATCAinsGTCG	p.Ile654_Ile655delinsValVal	n=3
*CDK4*		Amplification		n=5
*HRAS*		Amplification		n=3
* *	3	c.121C>T	p.Arg41Trp	n=1
* *	3	c.194G>T	p.Ser65Ile	n=1
*MYOD1*		Amplification		n=4
* *	1	c.122G>T	p.Arg41Leu	n=1
*CDKN2A*		Amplification		n=4
*TERT*	0	c.-143C>T (C247T)		n=1
* *	0	c.-124G>A (C228T)		n=1
*EGFR*		Amplification		n=2
*FBXW7*	10	c.1513C>G	p.Arg505Gly	n=1
* *	11	c.1805C>T	p.Thr602Ile	n=1
*FGFR1*		Amplification		n=2
*FGFR2*		Amplification		n=2
*FGFR3*		Amplification		n=2
*CTNNB1*	3	c.110C>T	p.Ser37Phe	n=1
*GNA11*	4	c.600C>G	P.Ile200Met	n=1
*NRAS*		Amplification		n=1
*RAF1*		Amplification		n=1

Nineteen out of the 49 (38.8%) patients with SCCA tested presented no alterations among the 42 screened genes. Most of them were HPV positive, with 89% (n=17/19) of HPV16 and 11% (n=2/19) of other HR-HPV. Forty-seven percent (n=9/19) of SCCA harbored integrated or mixed forms of HPV DNA, whereas 32% (n=6/19) harbored only episomal forms of oncogenic HPV DNA. On the contrary, 61.2% (n=30/49) patients with SCCA presented one alteration or more. A majority of them had an HPV16 infection (90%, n=27/30), one had an HPV16+33 infection and 2 were HPV negative. Almost 53% (n=16/30) had integrated or mixed forms, 26.6% (n=8/30) had an episomal HPV and 20% (n=6/30) were not identified. No difference was observed between the 2 groups concerning HPV status (p-values: 1 and 0.1581 between HPV positive/negative and HPV16/other HR-HPV respectively) nor integration status (p-value: 0.7397).

**Figure 3:**
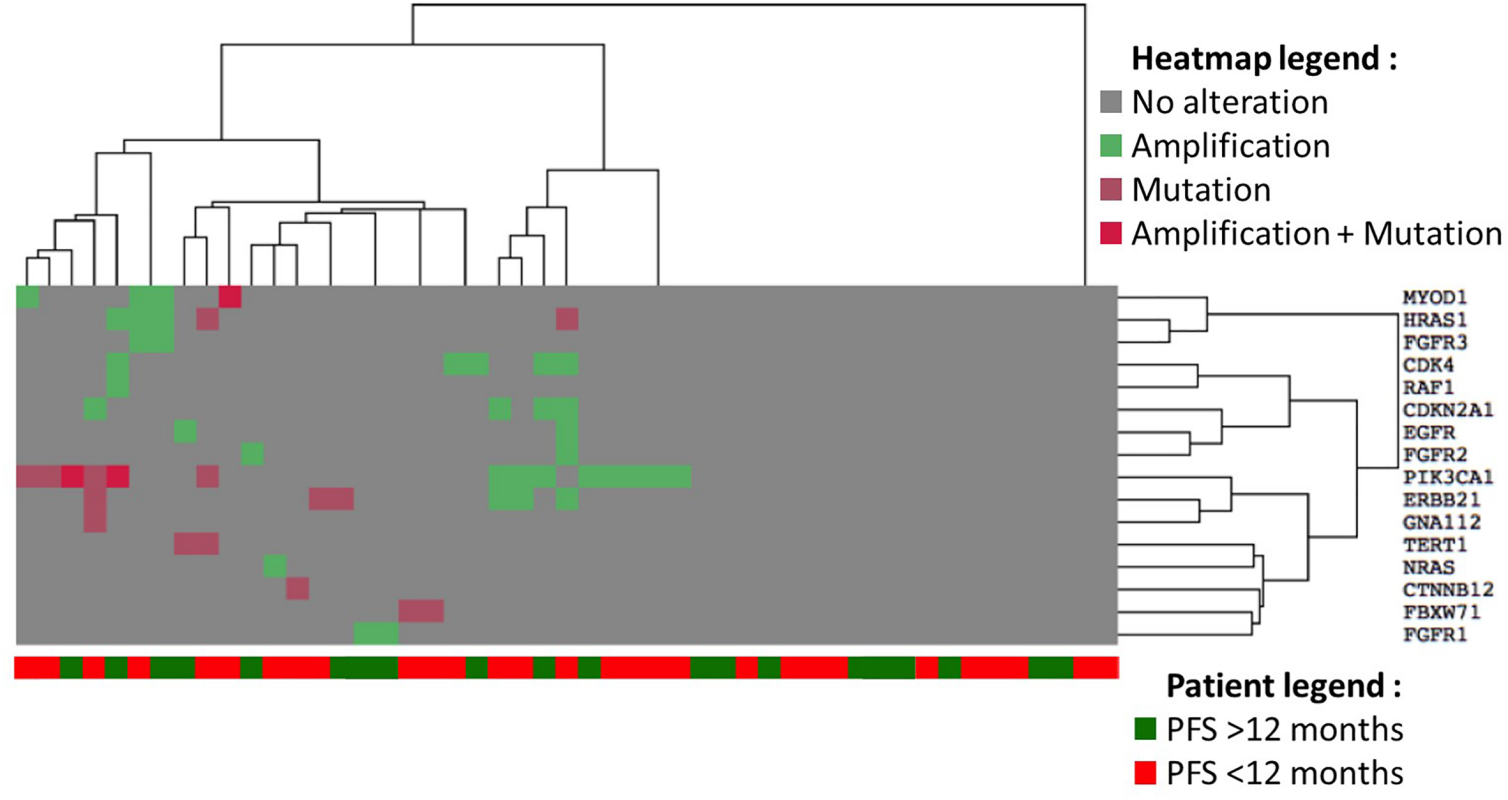
Heatmap of gene alteration frequency clustered by PFS (whether it reaches 12 months or not). The type of alterations is described in the heatmap legend, with the list of genes on the right, and PFS of patients is found at the bottom of the heatmap.

**Figure 4:**
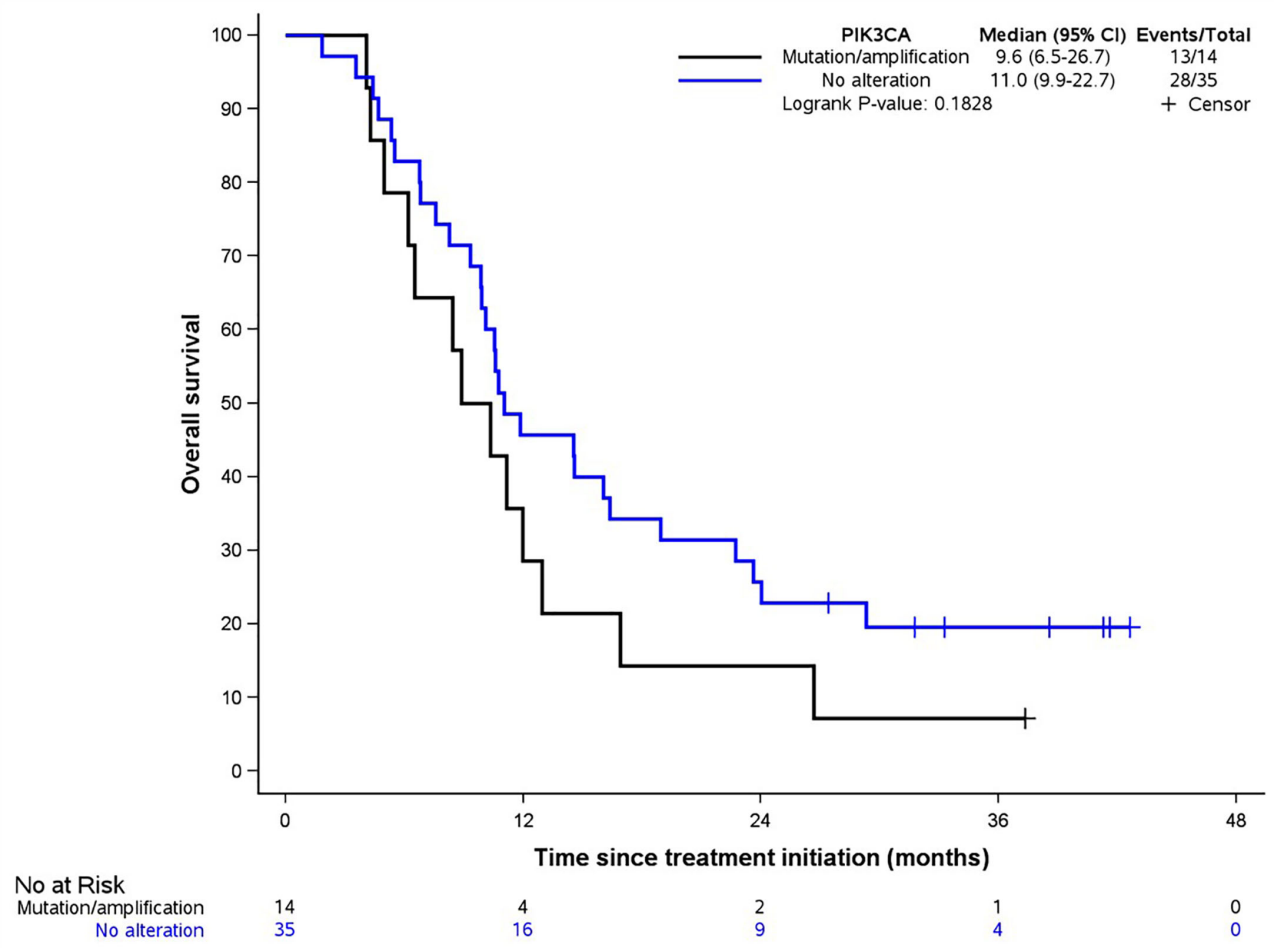
OS in presence of *PIK3CA* alteration. Blue line symbolizes no alteration, black line symbolizes the presence of a mutation and/or amplification.

A heatmap was realized to cluster genomic alteration occurrence to PFS (more or less than 12 months) (**Figure 3**; **Table 8**). There was clearly no aggregation of a genomic alteration subset with the probability to be progression free after 12 months of follow up. Since *PI3KCA* amplification and mutations were the most frequent, we assessed the influence of these genomic alterations on SCCA patient overall survival. No difference of OS was observed between patients harboring *PIK3CA* alterations or WT *PIK3CA* (**Figure 4**, p-value: 0.1828).

**Table 8:** Patient characteristics according to time of PFS.

	PFS<12 months	PFS>12 months	p-value
	n=29	n=20	0.1055
**Sexe**			
Female	n=23	n=18	0.4446
Male	n=6	n=2	
**HPV status**			
HPV16	n=27	n=18	1
Other HPV and HPV negative	n=2	n=2	
**Integration status**			
Episomal	n=6	n=8	0.0952
Integrated/mixed	n=18	n=7	
NR or HPV negative	n=5	n=5	
**TERT status**			
WT	n=28	n=19	1
Mutated	n=1	n=1	
**PIK3CA status**			
WT	n=19	n=16	0.3444
Altered	n=10	n=4	

## Discussion

Epitopes-HPV02 study was the first prospective clinical trial that included SCCA patients with advanced diseases and demonstrated the ability of Docetaxel-based polychemotherapy to induce long term remissions (15). An ancillary study was performed to analyze the distribution of HPV genotypes, integration status and oncogenic-related alterations in this population.

In our study, a majority of SCCA was HPV positive, mostly HPV16 which is the most frequent high risk type in this disease (24–26). Although HPV-negative SCCA are well-known for being associated with a poor outcome compared to their HPV-positive counterparts (24–26), no significant difference of PFS and OS between HPV-positive and -negative SCCA was observed in the present study, very likely due to the weak number (n=2) of HPV-unrelated samples contained in the present cohort.

Integration of HPV genome into the host genome has been shown to be correlated with disease progression in the context of both anal and cervical (pre-)cancers (21). However, it is important to notice that all HPV-mediated (pre-)cancers do not contain integrated forms of HPV. Indeed, while episomal HPV DNA is observed in the large majority (>90%) of low-grade squamous intraepithelial lesion, it can also be detected in up to 70% of high-grade intraepithelial lesions and invasive carcinoma (depending on both tumor stage and anatomical site) (27). E6 and E7 can be expressed in this case *via* the inhibition of the binding between E2 and its binding sites (E2BS) at the viral promoter due to the methylation of said E2BS (28). Several mechanisms of viral integration into host genome have been described in the literature (29) but, most often, HPV integration involves a break in the *E2* gene, leading to the overexpression of E6 et E7. Integrated/mixed forms were approximately twice more frequent (51% vs 28.6%) in our study compared to episomal forms. Of note, the percentage of “pure” episomal HPV DNA is likely to be slightly overestimated in this study given that, in a minority of cases, HPV could be integrated in host genome without *E2* disruption (these latter are actually only detectable by sequencing) (30). Despite this bias, the percentage of integrated/mixed forms in our study composed of relapsing and metastatic SCCA was similar to what can be observed in localized HPV-associated cancers (27). The better PFS observed in the case of patients harboring episomal HPV DNA raises the hypothesis of a predictive impact of HPV integration status on DCF efficacy in advanced SCCA. Further investigations should, however, be undertaken to confirm this observation.

Telomeres are nucleoprotein complexes playing a critical role in chromosome stability. Loss of telomere functions results in genetic instability and impairs cell viability. Telomeric complexes also participate to chromosome repair. Then, telomere nucleoprotein dysfunctions impaired DNA break repair capacities conferring to primary or cancer cells an enhanced sensitivity to ionizing radiation (31). The telomerase is the enzyme reconstituting telomeres and is constituted of several subunits in which the catalytic reverse transcriptase TERT is essential to telomerase activity, conferring *in fine* cellular immortalization by preventing replicative senescence. *TERT* promoter mutations are correlated to increased *TERT* expression and a worse prognosis as confirmed by a recent meta-analysis in glioma patients (32). *TERT* promoter mutations have been previously identified in glioblastoma (84%), urothelial carcinoma (64.5%), oligodendroglioma (70.0%), medulloblastoma (33.3%) and hepatocellular carcinoma (31.4%) (33). Of note, these mutations were not detected in most gastrointestinal cancers including gastric, cholangiocarcinoma and pancreatic cancers. The C228T and C250T mutations were the most frequent in *TERT* promoter [77.5% and 20.8% in glioma respectively (34)], inducing a consensus sequence bound by E-Twenty-Six (ETS) transcription factors (35). A similar distribution was observed in our study in *TERT* mutated SCCA wherein C228T and C250T were detected in 73% and 23.3% of the cases. However, *TERT* promoter mutation distribution remains scarce in SCCA, even in HPV negative cases where the absence of immortalization by E6 viral oncoprotein could have had an impact on mutational frequency of *TERT* promoter. Therefore, *TERT* promoter mutations are not correlated to HPV status, type, nor disease progression. As the EDITH V cohort, composed of localized SCCA, presented the same profile of *TERT* promoter mutation than the HPV02 cohort composed of metastatic SCCA, *TERT* promoter mutations also do not appear to be associated to SCCA status.

Furthermore, when analyzing somatic mutations in other genes, it appeared that SCCA were not highly mutated, the most frequently mutated being *PIK3CA*. This result was similar to others (36–41). In general, most common *PIK3CA* mutations are E542K and E545K, with 75% for the last one (37, 39). In our study we found 6 mutations in *PIK3CA*, all being E545K (1633G>A), which is mostly linked to an APOBEC (Apolipoprotein B mRNA editing enzyme catalytic polypeptide-like) alteration (42). As most substitutions (60%, n=6/10) retrieved in our study for all genes were C>T/G>A, it is possible that some of them may in part be due to the activity of APOBEC, creating “passenger mutations” opposed to “driver” to the oncogenesis. Indeed, it has been shown in Head and Neck Squamous Cell Carcinoma (HNSCC) that APOBEC activity and mutations are concordant between viral genome and host cell genome (43). We also found no *TP53* mutations in HPV-positive SCCA, as opposed to HPV-negative SCCA (44). Even if mutations were not associated with PFS and OS in our study, we observed a high number of gene amplifications (39 amplifications on tumors of 23 patients), mostly in *PIK3CA*. Gene amplification could be a consequence of HPV integration into the host genome, as shown elsewhere (45, 46), which could explain the high proportion of this alteration in the present study. Indeed, as HPV genome is integrated into host genome, regions flanking the viral genome are amplified at the same time as HPV genes in a rolling circle manner (47, 48). Six out of ten patients with a *PIK3CA* amplification presented integrated HPV forms, which was not statistically different, whereas patients with *CDK4* and *CDKN2A* amplifications presented integrated HPV forms in 5/5 and 3/4 cases respectively (p-values: 0.0079 and 0.485 respectively). The fact that HPV integration is sometimes located in or near amplification regions has been shown for other genes, like *MYC* (45), which is in favor of the impact of viral integration, in late stages of SCCA, on oncogenesis by deregulating oncogenes and mostly amplifying pro-oncogenes.

The main limitation of our study is its small sample size, and the HPV integration status analysis is based in 39 patients. However, to date, this clinical situation is in the scope of active clinical and translational research, and several prospective trials are ongoing (49). More robust confirmatory data are awaited in the near future.

Targeted therapy for patients with HPV-associated cancers resisting to standard treatments are ongoing. Bevacizumab (anti-VEGF antibody) and pembrolizumab (anti-PD1 antibody) were approved in progressive and metastatic cervical cancers (50), and were also evaluated in SCCA, as well as other anti-PD1 antibodies (nivolumab, retifanlimab) (51), and showed a promising result in a subgroup of patients (52–54).

In conclusion, as HPV status, integration status, *TERT* promoter mutations, and mutational profiling are not significantly correlated to PFS and/or OS in this study, DCF chemotherapy should be proposed to all SCCA patients failing radio-chemotherapy (CRT) or with metastatic disease. Further investigations are required to identify SCCA-related predictive biomarkers.

## Data availability statement

The raw data supporting the conclusions of this article will be made available by the authors, without undue reservation.

## Ethics statement

The studies involving human participants were reviewed and approved by The French ethic committee (NCT02402842). The patients/participants provided their written informed consent to participate in this study.

## Author contributions

CB, SK, J-LP and DG contributed to conception of the whole study or a part of it, and CB coordinated the clinical trial. AD, DG and PR performed the experiments and CB, AD, MH and AM performed data analysis and/or statistical analysis. AD wrote the first draft of the manuscript and CB, SK, AM and MH wrote sections of the manuscript. FM, LS, AV, AT, AL, ChlM and ChrM allowed data collection. All authors contributed to the article and approved the submitted version.

## Funding

Part of this work was funded by the Région Franche-Comté and the Belgian Fund for Scientific Research (FNRS; MIS F.4520.20).

## Acknowledgments

The authors would like to thank Guadalupe Inés Tizón for English writing assistance. We also thank the biobank ‘Tumorothèque Régionale de Franche-Comté’ (registration number BB-0033-00024) for biobanking. 

## Conflict of interest

CB has received a research grant from companies Bayer and Roche, and was an advisory board member of Bayer, MSD and Pierre Fabre companies. None of them had a role in the study design, analysis, or interpretation of the results.

The remaining authors declare that the research was conducted in the absence of any commercial or financial relationships that could be construed as a potential conflict of interest.

## Publisher’s note

All claims expressed in this article are solely those of the authors and do not necessarily represent those of their affiliated organizations, or those of the publisher, the editors and the reviewers. Any product that may be evaluated in this article, or claim that may be made by its manufacturer, is not guaranteed or endorsed by the publisher.
